# COVID-19 and Youth Psychopathological Distress in Umbria, Central Italy: A 2-Year Observational Study in a Real-World Setting

**DOI:** 10.3389/fpsyt.2022.869326

**Published:** 2022-05-19

**Authors:** Giulia Menculini, Giorgio Pomili, Francesca Brufani, Agnese Minuti, Niccolò Mancini, Martina D’Angelo, Sonia Biscontini, Enrico Mancini, Andrea Savini, Laura Orsolini, Umberto Volpe, Alfonso Tortorella, Luca Steardo

**Affiliations:** ^1^Section of Psychiatry, Department of Medicine and Surgery, University of Perugia, Perugia, Italy; ^2^Psychiatric Unit, Department of Health Sciences, University Magna Graecia of Catanzaro, Catanzaro, Italy; ^3^Mental Heath Department, Azienda Unità Sanitaria Locale (AUSL) Umbria 2, Terni, Italy; ^4^Comunità “La Tenda” Cooperativa Sociale, Foligno, Italy; ^5^Unit of Clinical Psychiatry, Department of Clinical Neurosciences/DIMSC, School of Medicine, Polytechnic University of Marche, Ancona, Italy

**Keywords:** adolescents, COVID-19, psychopathological distress, psychiatric disorders, young adults, youth mental health

## Abstract

**Introduction:**

Adolescents and young adults represent a vulnerable population in the context of the Coronavirus disease 2019 (COVID-19) pandemic. The present retrospective study aims to investigate the pandemic’s psychological impact on adolescents and young adults by analyzing data from an outpatient mental health service dedicated to youths in Umbria, central Italy.

**Materials and Methods:**

The clinical charts of subjects aged 14–24 who first accessed the service in the timeframe between March 1st, 2019, and February 28th, 2021, were reviewed. Subjects were divided into two subgroups according to the period of time when they accessed the service (pre-COVID-19 vs. during- COVID-19 outbreak). Bivariate analyses were performed using the Chi-square test and the Welch’s *t*-test. A secondary analysis was performed considering only subjects suffering from psychiatric disorders. Furthermore, data concerning individuals who were already followed by the service before the pandemic were analyzed by the McNemar’s test and the *t*-paired test to assess changes in treatment features.

**Results:**

The number of new accesses during the pandemic period remained stable. After the emergency onset, youths accessing the service showed a higher prevalence of anxiety disorders (*p* = 0.022). During the COVID-19 period, services were more frequently delivered by using a digital mental health approach (*p* = 0.001). Psychopharmacological treatment was more frequently prescribed among subjects that were referred to the service after the pandemic onset (*p* = 0.033). As for substance use, a highly significant reduction in opioid use was observed (*p* = 0.003). Family therapy was delivered less frequently in the during-COVID-19 subgroup, especially in the subpopulation of subjects suffering from psychiatric disorders (*p* = 0.013). When considering subjects referred to the service in the pre-COVID-19 period, the number of interventions provided to this population increased after the pandemic outbreak (*p* = 0.038).

**Conclusion:**

In the context of the COVID-19-related public health crisis, youths represent an at-risk population for which pathways to care should be reinforced, and targeted interventions, including psychosocial treatments, should be implemented.

## Introduction

The Coronavirus disease 2019 (COVID-19) pandemic represents an unprecedented health emergency affecting healthcare systems worldwide, with serious socioeconomic consequences (1). In the complex scenario that arose from the infection spread, mental health was prioritized because of the high psychological distress caused by social distancing and isolation (2, 3). Indeed, an increasing prevalence of anxiety, depression, irritability, and insomnia has been documented among the general population after the COVID-19 outbreak (4). The COVID-19 pandemic hit Italy consistently, and data from the epidemiological register of the Umbria region, in the center of the country, also reported a dramatic increase in the curve since the beginning of the pandemic spread (5). The rise in contagions forced the regional council to adopt severe restrictive measures to preserve the proper functioning of the hospital and healthcare network, with high psychopathological distress for the population. Indeed, previous reports underlined that the mental health of the general population in central Italy was significantly impacted by the pandemic, as also demonstrated by the increase of psychiatric consultations in emergency services (6).

However, the risk of developing COVID-19-related psychological distress was particularly high in vulnerable populations, such as in subjects aged 16–24 years old, who represented one of the groups most affected by the pandemic emergency (7–10). Lockdown measures had an unfavorable impact on adolescents and young adults, and a large amount of literature highlighted a greater risk for the occurrence of psychiatric symptoms due to a change in their lifestyles and habits (11) as well as lower levels of post-traumatic growth (12). For instance, the closure of schools has imposed distance learning as an alternative to maintain continuity in the education of children and adolescents (13). The subsequent prolonged social isolation threatened the psycho-physical wellbeing of youths, worsening or unmasking psychopathology (14). During the pandemic, there has been a widespread increase in depression, anxiety, irritability among children, and adolescents, and suicidal behaviors (8, 15, 16). Studies conducted in Italy already demonstrated a high prevalence of moderate or severe anxiety among youths (17), as well as a higher risk for developing problematic internet use in this population during the pandemic (18).

Furthermore, limitations due to infection imposed an adaptation in the availability of psychiatric and psychosocial interventions in dedicated settings, which were pointed out as critical needs for this population (19–21). For this reason, implementing telepsychiatry and integrating interventions to maintain regular and emergency child and adolescent psychiatric treatment during the pandemic was identified as a significant challenge that could be necessary for limiting long-term consequences on mental health (22). In fact, integrated intervention programs (medical intervention, psychotherapy, psychoeducation to family members, social intervention) seem to have a considerably better impact than treatment-as-usual in the youth population, especially at disease onset (23).

Several countries had already allocated tailored funding for the mental health of adolescents and young adults before the pandemic (24) and conducted specific campaigns to address children and young people’s mental health in the COVID-19 era (25). To this end, the European Year of Youth 2022 presents an opportunity for countries and organizations to enhance health promotion initiatives and focus on mitigating mental health problems in this population (26).

Within this scenario, the Italian Umbria region had already decided to allocate specific funds to widen the possibility of mental health departments supporting youths with psychopathological distress. The Addiction Service (SerD) of Local Mental Health 2 (USL Umbria 2) in Foligno agreed with the Umbria region to expand its curative offer by implementing an outpatient service dedicated to adolescents and young adults who present psychological distress.

Based on these premises, the present study aimed to investigate the pandemic’s impact on adolescents and young adults, analyzing data from the abovementioned outpatient youth mental health service. Notably, changes in access to mental health care, clinical and treatment features of patients in charge of the service before the pandemic were furtherly analyzed. A secondary analysis was performed to evaluate access to care and changes in clinical and treatment features of subjects suffering from psychiatric disorders. Particularly, we expect to detect significant changes in diagnostic and treatment (both psychopharmacological and psychosocial) features after the COVID-19 outbreak, possibly reflecting differences in pathways to care and patterns of care for this population.

## Materials and Methods

### Study Procedures

The present study was performed by carrying out a retrospective chart review analysis of clinical data collected during the time period between March 1st, 2019 and February 28th, 2021, at the Addiction Service (SerD) of Local Mental Health 2 (USL Umbria 2) in Foligno, Umbria, Italy. Clinical charts of subjects aged 14–24 who first accessed the service between March 1st, 2019, and February 28th, 2021, were retrospectively reviewed. In the study, we included both subjects who had a personal history of substance use disorders (SUD) and subjects who reported no history of SUD. Indeed, SUD can also be considered an early sign of psychological distress in youths and may represent a “red flag” for the later development of clear-cut psychiatric symptoms (27).

Information concerning the personal and clinical history of the included subjects was extracted from the electronic medical charts achieved from the online platform PoInT GeDi (28). Data were inserted in two electronic datasets created *ad hoc* for the current project. In the first dataset, subjects who first accessed the service between March 1st, 2019, and February 28th, 2021, were entered. This population was then divided in two subgroups, namely subjects who had accessed the service for the first time in the period March 1st, 2019, and February 28th, 2020 (pre-COVID-19 period) and those who referred to the service for the first time in the period March 1st, 2020 and February 28th, 2021 (during-COVID-19 period). Indeed, the national lockdown was established in Italy on March 9th, 2020, which also concerned the Umbria region. Furthermore, the first case of COVID-19 was confirmed in Italy at the end of February 2020, and an increase in COVID-19 cases and related hospitalizations registered in Umbria at the beginning of March (29). This dataset was used to compare socio-demographic, clinical, and treatment characteristics among the two populations to evaluate significant differences between subjects who accessed the service before and after the pandemic outbreak and between the treatments provided in the two populations in the two different periods. Socio-demographic data collected for the included subjects were age, gender, nationality, marital status, scholarity, working status, and living status. As for clinical information, data concerning medical comorbidities, SUD (alcohol, amphetamines, cannabinoids, cocaine, and heroin), psychiatric diagnosis, suicide attempts, and non-suicidal self-injurious behavior was collected. Moreover, we extracted the following treatment-related features: current psychopharmacological treatment (antidepressants, antipsychotics, benzodiazepines, mood stabilizers), replacement treatment for addiction, other pharmacological treatments, psychosocial interventions (individual psychotherapy, family therapy, social, and educational interventions), and treatment in a residential facility.

Only subjects who first accessed the service in the pre-COVID-19 period were considered in the second dataset. Information concerning treatments provided before and after the pandemic was collected to analyze significant changes in treatment features possibly related to the COVID-19 pandemic. Data concerning clinical characteristics and treatment features (see above) was collected for both pre- and during-pandemic periods.

To address the secondary aim of the study, a further analysis was performed on a subsample of subjects suffering from psychiatric disorders. The following nosographic entities were considered: schizophrenia spectrum disorders, depressive disorders, bipolar disorders, anxiety disorders, impulse control disorders, neurodevelopmental disorders, substance-related disorders, personality disorders, and adjustment disorders. Trained psychiatrists and psychologists with specific expertise on youth mental health carried out the diagnostic evaluation by using the Diagnostic and Statistical Manual of Mental Disorders, 5th edition (DSM-5) (30). Subjects affected by neurocognitive disorders or medical illnesses that might significantly influence mental health status were excluded. The analysis was then repeated following the aforementioned procedure, focusing on this subsample.

According to the study’s observational nature, all the included subjects underwent treatment as usual. The study was conducted in accordance with Declaration of Helsinki and followed the Good Clinical Practice Guidelines. All the included subjects signed their informed consent for having their data used for research purposes. In the case of minors, informed consent was also obtained by parents or those who exercised parental authority. The study protocol was approved by the Ethics Committee of Umbria Region (protocol N° 23369/21/ON).

### Study Setting

The facility where the study was carried out is a multidisciplinary service aimed at providing health promotion, prevention, and treatment of people with problems of addiction to legal or illegal psychoactive substances (drugs, alcohol, smoking), or addiction without the use of substances (e.g., gambling and video addiction). The service is equipped with specific facilities dedicated to youth suffering from psychological distress, with separate pathways for those who report SUD, including alcohol, and those who do not. Indeed, despite the service itself being dedicated to addiction problems, the growing number of youths reporting mental health problems led to the institution of a specific project addressed to young people with psychological distress without SUD comorbidity. This path of care is part of two specific projects, named “Girovento” and “Giovani 2.0.” These projects attempt to address the need for new clinical-organizational responses concerning increasingly complex requests coming from youths in the fourteen-twenty-four age group; all these treatment pathways operate in close integration with the services of child and adolescent psychiatry, inpatient, and outpatient community mental health services for adults, and with the school psychologists consulting service, as well as with social services.

The multidisciplinary team discusses weekly the clinical cases of subjects who access these projects, identifying and elaborating individualized therapeutic projects, e.g., individual psychotherapy, family therapy, social and educational interventions, peer groups and support groups addressed to parents. Psychiatric evaluation and treatment are also provided whenever needed.

### Statistical Analysis

A descriptive analysis of socio-demographic, clinical, diagnostic, and treatment features was performed to evaluate the distributional properties of the variables in the study sample. Categorical variables were expressed as frequencies, while continuous variables were expressed as mean and standard deviation (SD) or median and interquartile range (IQR) according to the normality of the distribution. The normality of continuous variables was verified by using the Kolmogorov-Smirnov test.

Bivariate analyses were carried out to compare the subgroups of subjects who accessed the service before and after the pandemic outbreak. We performed the Chi-Square test for categorical variables since levels of the variables were mutually exclusive and the compared groups were independent. All tests were performed for 2 × 2 cross tabs. The assumption according to which the expected cell count should be ≥ 5 in at least 80% of the cells was verified for all tests, and when this assumption was not met the Fisher’s exact test was carried out (31, 32). The Welch’s *t*-test was used for continuous variables due to the presence of outliers. The analysis was conducted using a parametric test due to the sensitivity of this technique, which guarantees sufficient robustness in case of normality assumption violation in sample sizes like the one we considered (33–35). In order to assess differences concerning the pre- and during-COVID-19 period for subjects who first accessed the service before the pandemic break, the Mc Nemar’s test was used for categorical variables since we attempted to find statistically significant differences in paired variables categorized as dichotomous. The sample consisted of all youths accessing the service, and since no restrictions in access to the service were established (e.g., presentation modalities, area of residence), the sample could be considered as representative of the population of interest (youths accessing to care in the Umbria region). The Student’s *t*-test for paired samples was employed for continuous variables. We chose not to apply a *p*-value correction (e.g., Bonferroni correction) to avoid type II errors. Indeed, we conducted exploratory analyses for testing a hypothesis mainly concerning two groups of variables, namely diagnostic and treatment features, and thus did not want to miss possible significant association worthy of being further explored (36). All *p*-values were two-tailed. Since the reporting of results according to a continuous approach rather than to a fixed threshold (e.g., *p* < 0.05) has been largely advocated (37, 38), findings from the present study will be presented in terms of high (*p* < 0.01), medium (*p* < 0.05 and ≥ 0.01), and low (*p* < 0.1 and ≥ 0.05) significance. All analyses were performed using the Statistical Package for Social Sciences (SPSS) version 26 for Windows Inc. (Chicago, IL, United States).

## Results

### Description of Sample Characteristics

The overall study population consisted of 110 subjects, with a higher prevalence of male gender (*n* = 77, 70%) and a median age of 19 ± years old (IQR 5, range 14–24). Most subjects in the sample were Italian (*n* = 97, 88.2%). None of the included subjects was married, and the majority lived with their family of origin (*n* = 77, 70%). As for working status, 56 (50.9%) were current students, whilst 19 (17.3%) did not study or work. In the sample, 57 (51.8%) youths were referred to the service before the COVID-19 pandemic outbreak and 53 (48.2%) had their first contact after the pandemic spread. The most frequently reported addictive behavior was alcohol use (*n* = 35, 31.8%), followed by cannabis use (*n* = 31, 28.2%).

Subjects suffering from a psychiatric disorder were 79 (71.8%), among which 48 (60.8%) were males. The median age in the sample was 18 (IQR 4) years old, ranging from 14 to 24. When analyzing the period when youths referred to the service, 42 (53.2%) accessed the outpatient facility before the COVID-19 outbreak. Most subjects in this subsample lived with their family of origin (*n* = 65, 82.3%), while a minority of them lived in residential facilities (*n* = 7, 8.9%) or on their own (*n* = 6, 7.6%). At the time of clinical assessment, 53 subjects (67.1%) were students, while 14 (17.7%) did not study or work. In this subsample, the most common psychiatric disorders were represented by anxiety disorders (*n* = 37, 46.8%) and adjustment disorders (*n* = 22, 27.8%). Concerning addictive behaviors, most subjects reported cannabis use (*n* = 29, 36.7%) (see Tables 1, 2). Six (7.6%) subjects presented self-aggressive behaviors, and one attempted suicide. Psychopharmacological treatment was prescribed in 21 (19.9%) subjects in the overall sample. Particularly, antipsychotics were prescribed to 10 (9.1%) subjects, while five (4.5%) received antidepressants, 10 (9.1%) took mood stabilizers, and seven (6.4%) underwent anxiolytics prescription. As for psychosocial interventions, individual psychotherapy was delivered to 72 (65.5%) subjects, and family therapy was provided in 17 (15.5%) cases. Moreover, 49 (44.5%) and 20 (18.2%) subjects underwent social-educational interventions.

**TABLE 1:** Comparison of socio-demographic and clinical characteristics of subjects accessing the service before (pre-COVID-19; *n* = 57, 51.8%) and after the COVID-19 pandemic outbreak (during-COVID-19; *n* = 53, 48.2%).

Socio-demographic and clinical characteristics					
	**Pre-COVID-19 (*n*,%)**	**During-COVID-19 (*n*,%)**	χ **^2^**	***p***	**OR (95% CI)**
Female gender	19 (33.3)	14 (26.4)	0.340	0.560	0.718 (0.315–1.634)
Italian nationality	50 (87.7)	47 (88.7)	0.000	1.000	1.097 (0.343–3.501)
Unemployed	9 (15.8)	10 (18.9)	0.030	0.862	1.240 (0.461–3.338)
Living with family of origin	35 (61.4)	42 (79.2)	3.357	*0.067*	2.400 (1.024–5.624)
Living alone	5 (8.8)	2 (3.8)	0.465	0.495	0.408 (0.076–2.199)
Living in a residential facility	13 (35.1)	7 (13.7)	0.000	1.000	0.849 (0.215–3.346)
Referral to a residential facility	3 (5.3)	0 (0)	1.227	0.244	0.505 (0.418–0.609)
NSSI	4 (7)	2 (3.8)	0.108	0.680	0.520 (0.091–2.961)
Drop-out	17 (29.8)	15 (28.3)	0.000	1.000	0.929 (0.407–2.118)

	**Pre-COVID-19 (mean, *SD*)**	**During-COVID-19 (mean, *SD*)**	**Welch’s *t*-test**	***p***	

Age	18.53 (2.82)	19.77 (2.63)	5.758	***0.018***	
Number of interventions	30.89 (37.63)	22.89 (33.66)	1.596	0.209	

**Diagnostic features**					

	**Pre-COVID-19 (*n*,%)**	**During-COVID-19 (*n*,%)**	χ **^2^**	***p***	**OR (95% CI)**

Psychiatric comorbidity	37 (64.9)	42 (79.2)	2.124	0.145	2.064 (0.875–4.869)
Adjustment disorders	12 (21.1)	10 (18.9)	0.002	0.962	0.872 (0.342–2.227)
Anxiety disorders	13 (22.8)	24 (45.3)	5.249	***0.022***	2.801 (1.231–6.371)
Bipolar disorders	0 (0)	0 (0)	−	−	−
Depressive disorders	6 (10.5)	6 (11.3)	0.000	1.000	1.085 (0.327–3.599)
Impulse control disorders	8 (14)	13 (24.5)	1.337	0.248	1.991 (0.751–5.276)
Neurodevelopmental disorders	3 (5.3)	3 (5.7)	0.000	1.000	1.080 (0.208–5.600)
Personality disorders	8 (14)	8 (15.1)	0.000	1.000	1.089 (0.377–3.144)
PTSD	2 (3.5)	1 (1.9)	0.000	1.000	0.529 (0.047–6.008)
Schizophrenia spectrum disorders	3 (5.3)	0 (0)	1.227	0.244	0.505 (0.418–0.609)
More than one psychiatric disorder	14 (24.6)	17 (32.1)	0.440	0.507	1.450 (0.630–3.341)
SUD	36 (63.2)	33 (62.3)	0.066	0.797	0.840 (0.393–1.795)
Alcohol use disorders	18 (31.6)	17 (32.1)	0.000	1.000	1.023 (0.458–2.284)
Cannabis use disorders	13 (22.8)	18 (34)	1.182	0.277	1.741 (0.751–4.033)
Cocaine use disorders	4 (7)	5 (9.4)	0.013	0.736	1.380 (0.350–5.440)
Opioid use disorders	13 (22.8)	1 (1.9)	9.020	***0.003***	0.065 (0.008–0.517)
More than one SUD	7 (12.3)	6 (11.3)	0.000	1.000	0.912 (0.286–2.911)

**Treatment features**					

Psychopharmacological treatment	6 (10.5)	15 (28.3)	4.526	***0.033***	3.355 (1.191–9.452)
Antidepressants	0 (0)	5 (9.4)	3.669	***0.023***	0.457 (0.371–0.563)
Antipsychotics	4 (7)	6 (11.3)	0.205	0.517	1.691 (0.450–6.362)
Benzodiazepines	3 (5.3)	4 (7.5)	0.010	0.709	1.469 (0.313–6.896)
Mood stabilizers	2 (3.5)	8 (15.1)	3.169	***0.047***	4.889 (0.988–24.185)
Alcohol substitution therapy	1 (1.8)	1 (1.9)	0.000	1.000	1.077 (0.066–17.663)
Opioid substitution therapy	10 (17.5)	1 (1.9)	5.942	***0.016***	0.090 (0.011–0.733)
Educational interventions	9 (15.8)	11 (20.8)	0.183	0.669	1.397 (0.528–3.697)
Family therapy	13 (22.8)	4 (7.5)	3.796	*0.051*	0.276 (0.084–0.910)
Individual psychotherapy	36 (63.2)	36 (67.9)	0.105	0.745	1.235 (0.561–2.719)
Social interventions	28 (49.1)	21 (39.6)	0.656	0.418	0.680 (0.319–1.449)
Digital interventions	2 (3.5)	15 (28.3)	11.092	***0.001***	10.855 (2.345–50.244)

*NSSI, Non-suicidal self-injury; PTSD, Post-traumatic stress disorder; SUD, Substance use disorder. For all categorical variables, “yes” are listed. High (p < 0.01) and medium significance (p < 0.05 and ≥ 0.01) is reported in bold and italics, low significance (p < 0.1 and ≥ 0.05) is reported in italics. Data concerning the whole sample of youths referring to the service in the two considered periods are reported in this table.*

**TABLE 2:** Comparison of socio-demographic and clinical characteristics of subjects accessing the service before (PSY-pre-COVID-19; *n* = 37, 46.8%) and after the COVID-19 pandemic outbreak (PSY-during-COVID-19; *n* = 42, 53.2%).

Socio-demographic and clinical characteristics					
	**PSY-pre-COVID-19 (*n*,%)**	**PSY-during-COVID-19 (*n*,%)**	χ ^2^	***p***	**OR (95% CI)**
Female gender	17 (45.9)	14 (33.3)	0.837	0.360	0.588 (0.237–1.463)
Italian nationality	34 (91.9)	37 (88.1)	0.034	0.717	0.653 (0.145–2.941)
Unemployed	6 (16.2)	8 (19)	0.001	0.973	1.216 (0.379–3.898)
Living with family of origin	32 (86.5)	33 (78.6)	0.390	0.533	0.573 (0.173–1.895)
Living alone	4 (8.8)	2 (4.8)	0.345	0.411	0.413 (0.071–2.395)
Living in a residential facility	3 (8.1)	4 (9.5)	0.000	1.000	1.193 (0.249–5.716)
Referral to a residential facility	3 (8.1)	0 (0)	1.668	*0.098*	0.447 (0.348–0.574)
NSSI	4 (10.8)	2 (4.8)	0.345	0.411	0.413 (0.071–2.395)
Drop-out	5 (13.5)	10 (23.8)	0.769	0.381	2.000 (0.615–6.509)

	**PSY-pre-COVID-19 (mean, *SD*)**	**PSY-during-COVID-19 (mean, *SD*)**	**Welch’s *t*-test**	***p***	

Age	17.35 (2.47)	19.29 (2.62)	11.353	***0.001***	
Number of interventions	33.43 (34.45)	27.55 (36.40)	0.755	0.388	

**Treatment features**					

	**PSY-pre-COVID-19** (*n*,%)	**PSY-during-COVID-19** (*n*,%)	χ ^2^	***p***	**OR (95% CI)**

Psychopharmacological treatment	6 (16.2)	15 (35.7)	2.898	*0.074*	2.870 (0.977–8.437)
Antidepressants	0 (0)	5 (11.9)	2.909	*0.057*	0.500 (0.398–0.628)
Antipsychotics	4 (10.8)	6 (14.3)	0.015	0.743	1.375 (0.356–5.306)
Benzodiazepines	3 (8.1)	4 (9.5)	0.000	1.000	1.193 (0.249–5.716)
Mood stabilizers	2 (5.4)	8 (19)	2.192	*0.094*	4.118 (0.815–20.802)
Alcohol substitution therapy	1 (1.8)	1 (1.9)	0.000	1.000	0.878 (0.053–14.551)
Opioid substitution therapy	10 (17.5)	1 (1.9)	5.942	***0.044***	0.440 (0.341–0.568)
Educational interventions	8 (21.6)	10 (23.8)	0.000	1.000	1.133 (0.394–3.259)
Family therapy	13 (35.1)	4 (9.5)	6.100	***0.013***	0.194 (0.057–0.666)
Individual psychotherapy	34 (91.9)	36 (85.7)	0.258	0.490	0.529 (0.123–2.287)
Social interventions	24 (64.9)	20 (47.6)	1.724	0.189	0.492 (0.199–1.219)
Digital interventions	1 (2.7)	15 (35.7)	11.307	***0.001***	20.000 (2.487–160.865)

*NSSI, Non-suicidal self-injury. For all categorical variables, “yes” are listed. High (p < 0.01) and medium significance (p < 0.05 and ≥ 0.01) is reported in bold and italics, low significance (p < 0.1 and ≥ 0.05) is reported in italics. Data concerning youths suffering from a psychiatric disorder as diagnosed according to the DSM-5 criteria periods are reported in this table.*

### Socio-Demographic and Clinical Characteristics of Subjects Accessing the Service Before and During the COVID-19 Pandemic

When comparing subjects accessing the service before (*n* = 57, 51.8%) and during the COVID-19 pandemic (*n* = 53, 48.2%), no differences in socio-demographic characteristics were found regarding gender and occupation. Subjects accessing the service after the pandemic started were older than those referred before the COVID-19 outbreak (mean age 19.77 ± 2.63 vs. 18.53 ± 2.82), and more often lived with their family of origin (79.2% vs. 61.4%), respectively with a medium (*p* = 0.018) and low (*p* = 0.067) significance.

When assessing addictive behaviors in the two subgroups, no differences were detected between subjects accessing the service before and during the COVID-19 pandemic except for opioid use, which was highly more prevalent in the pre-pandemic sample (22.8% vs. 1.9%, *p* = 0.003). Furthermore, youths accessing the service after the emergency onset showed a higher prevalence of anxiety disorders (45.3% vs. 22.8%, *p* = 0.022).

The two subgroups did not differ in the number of psychiatric visits and psychosocial interventions supplied. After the COVID-19 outbreak, services were more frequently delivered using a digital mental health approach (28.3% vs. 3.5%), with a high significance of the result (*p* = 0.001). No significant differences were detected in terms of drop-out rates.

A medium significance was found for differences in the prescrition of psychopharmacological treatment, which was more frequently prescribed among subjects that were referred to the service after the pandemic (28.3% vs. 10.5%, *p* = 0.033). Particularly, antidepressant and mood stabilizer prescription rates were higher after the COVID-19 outbreak (9.4% vs. 0%, *p* = 0.023; 15.1% vs. 3.5%, *p* = 0.047). When assessing changes in the delivery of psychosocial interventions, we evidenced a reduction in family therapy in the during-COVID-19 group (7.5% vs. 22.8%), with a low significance (*p* = 0.051). For comparison between subjects accessing the service before and after the pandemic spread, see Table 1.

When analyzing the secondary outcome of the study, by comparing subjects affected by psychiatric disorders referring to the service before (*n* = 37, 46.8%) and during (*n* = 42, 53.2%) the- COVID-19 pandemic (see Table 2), the only socio-demographic characteristic that differed among the two subgroups with a high significance was the age. Indeed, subjects accessing psychiatric services after the pandemic were older than those who were referred to the service before the infection outbreak (19.29 ± 2.62 vs. 17.35 ± 2.47, *p* = 0.001). Diagnostic features did not differ between the two subgroups, nor did the other clinical characteristics investigated in the present study.

Digital social and educational interventions and telepsychiatry interventions were significantly more frequent in the during-COVID-19 subgroup (35.7% vs. 2.7%, *p* = 0.001). Treatment prescription varied among the two populations with a low significance. Particularly, higher psychopharmacological prescription rates (35.7% vs. 16.2%, *p* = 0.074), especially for what concerned antidepressants (11.9% vs. 0%, *p* = 0.057) and mood stabilizers (19% vs. 5.4%, *p* = 0.094), were highlighted in the during-COVID-19 population.

Family therapies were less frequently administered to subjects who accessed the service after the pandemic spread when evaluating psychosocial interventions (9.5% vs. 35.1%), with a medium significance (*p* = 0.013). Moreover, none of the subjects accessing the service after the COVID-19 outbreak was referred to residential facilities, with a low significance when compared to those accessing the service before (0% vs. 8.1%, *p* = 0.098).

### Differences in Treatment Features During the COVID-19 Pandemic

Among subjects referred to the service before the pandemic, 18 (31%) dropped out of the therapeutic program before the COVID-19 spread. Only subjects who did not drop-out before the pandemic outbreak were considered for this sub-analysis (*n* = 39).

Medium significance was found in the difference between the number of interventions supplied before and during the COVID-19 pandemic (70.80 ± 107.117 vs. 34.55 ± 39.08, *p* = 0.038). Digital mental health services demonstrated a highly significant increase in the considered population (5.1% vs. 56.4%, *p* < 0.001). The rates of psychosocial interventions did not differ when comparing the period before and after the pandemic spread, and neither did psychopharmacological treatment features.

## Discussion

After the COVID-19 outbreak, there was a significant increase in the number of interventions supplied to subjects who first accessed the service in the “pre-COVID-19” period. We found an increase in the mean age of subjects who accessed the service in the “during-COVID-19” period, a higher prevalence of anxiety disorders, and an increase in the use of anxiolytics and mood stabilizers. A reduction in the prevalence of opioids use disorder and in the use of substitution therapy for opioid dependence was also observed. Furthermore, we observed a highly significant increase in digital mental health interventions in the “during-COVID-19” period, as well as a decrease in family therapies, both provided by digital tools and in-person, with a high significance of the difference in the subpopulation of subjects suffering from psychiatric disorders. Treatment features of people who were already followed by the service before the COVID-19 outbreak did not significantly change, except for the number of supplied interventions and the already mentioned increase of digital interventions.

New accesses to the service after the COVID-19 outbreak were stable, which is in line with the literature on the topic. Indeed, previous studies highlighted that the number of admissions to psychiatric care facilities showed trends similar to the pre-COVID-19 outbreak period (39). This result could be due to an adaptation of mental services to give help during the pandemic, especially implementing digital mental health services (40), while it is in contrast with other findings, e.g., those concerning the decrease of new accesses to psychiatric emergency units (41–43). Based on the stated above, access to care during the COVID-19 emergency should be further investigated since it represents a complex issue that relies on several possible determinants (44). Future research on the topic should thus consider these determinants, such as socio-economic factors (45, 46).

Our study also observed a significant increase in the number of follow-up interventions of subjects that were already being treated in the “pre-COVID-19” period. This has been made possible by the highly significant increase of digital mental health interventions (47–51). Indeed, digital mental health interventions, such as those delivered *via* mobile and web-based platforms, offer the potential to improve access to care while avoiding many existing barriers to receiving face-to-face intervention, including stigma and time (52–54). The evidence base for digital mental health interventions in the general population is rapidly accumulating (55, 56), and many studies on the topic reported that such interventions were either effective or partially effective in producing beneficial changes in the main psychological outcome variables, also among youngsters (50, 57, 58).

Dropouts from the therapeutic project did not face a statistically significant increase and were similar to those detected in studies conducted on similar populations before the pandemic spread (59). Despite this, an increasing trend in dropouts was evidenced after the COVID-19 outbreak and a positive, strong association was highlighted in the psychiatric disorders’ subgroup. To our best knowledge, literature concerning drop-out rates from outpatient psychiatric services during the pandemic is scant, especially for the youth population. Data from the present research are thus expected to be further clarified by future prospective studies, since adequate access to care represents a crucial issue in the field of early intervention (60).

The results have shown an increase of medium significance in the mean age of subjects accessing the service in the “during-COVID-19” period. We hypothesized that this finding could be due to better social support given by belonging to a group, such as schoolmates for adolescents, representing a protective factor against loneliness that can lead to anxiety and depressive symptomatology (61). Therefore, having finished school, with a consequent reduction in the sense of belongingness, may have a synergistic effect with the isolation linked to the pandemic and lockdown measures themselves. Many studies reported that loneliness threatens mental health (7, 62), leading to sleep disturbances and increased inactivity (63, 64). Greater severity of depressive symptomatology may also had been caused by loneliness, along with poor self-perceived overall health quality, impaired functional status, and a perceived negative change in the quality of life (65).

Concerning people who first sought help to the service after the COVID-19 outbreak, we found a higher prevalence of anxiety disorders in this population when compared to those referred before the pandemic spread. An increase in prescriptions of antidepressants and mood stabilizers was also highlighted, both for subjects suffering from psychiatric disorders and those who did not. Interestingly, the significance of the phenomenon was higher in the second group. Several studies confirmed our findings by detecting the increase in the prevalence of anxiety and depressive disorders in young adults during the COVID-19 pandemic (66, 67). Scientific papers that have evaluated the differences in the prescriptions of psychopharmacological treatments during the pandemic are scant. However, some studies showed an increasing trend (68). It should also be noted that the choice of pharmacological treatments in youth populations represents a critical issue, as demonstrated by the high prescription rates of off-label treatments in this population (69). In our sample, when a clear-cut diagnosis according to the DSM-5 criteria was not possible to be performed, pharmacological treatments were based on symptom dimensions. Indeed, the absence of a full-blown diagnosis does not necessarily mean the absence of an at-risk state, namely a totipotent condition that could hesitate in different exit syndromes (70–72). This could also explain higher rates of mood stabilizer prescription in the sample, even though the diagnosis of bipolar disorders did not significantly change. To this extent, it should be noted that the emergence of SUD or anxiety symptoms during youth may be the expression of a bipolar diathesis in young people, and this may partially explain the higher mood stabilizer prescription rate (73). Furthermore, we should consider that adjustment disorders may also manifest with disturbed conduct, which may more frequently benefit from mood stabilizers or antipsychotics in youths (74, 75). Due to the risk of dependence associated with benzodiazepines assumption, especially in a population of subjects accessing an addiction service, low-dose atypical antipsychotics and mood stabilizers were preferred for anxiety symptoms or anxiety disorders (76, 77).

As demonstrated by some reviews (10, 78), the COVID-19 pandemic and the lockdown measures may have negatively impacted youths’ mental health. First, school closure may have significantly impacted children and adolescents, particularly those aged between 5 and 18 (79). Lack of regular contact with friends may more frequently result in loneliness during adolescence and is not necessarily mitigated using phones or other communication forms (80). This context predisposes adolescents to psychopathological vulnerability, leading to an increasing trend in diagnoses of depressive and anxiety disorders (81). Accordingly, the prevalence of depression in young people across studies conducted in this period ranged from 22.6% to 43.7%, according to previous studies (66, 67), and an increase in the severity of pre-existing depression was detected (82). A survey conducted in China among 8,079 adolescents aged 12–18 revealed a high prevalence of symptoms of depression (43%), anxiety (37%), and combined depression and anxiety (31%) during the COVID-19 pandemic (83). Several risk factors, such as relatives suffering from COVID-19, were identified for the development of affective symptoms (84). These findings are considerable since youths suffering from psychiatric disorders represent an extremely vulnerable population, among which significant consequences could also emerge after the pandemic outbreak (85).

As expected, we observed a highly significant increase in telepsychiatry interventions in line with a large amount of literature. Several papers highlighted an increase in the prevalence of digital mental health interventions in young adults during the COVID-19 pandemic, and different psychological interventions were adapted to the online form (86, 87). We highlighted a decreasing trend in family therapies, with higher significance in the subgroup of subjects suffering from psychiatric disorders. Family therapies are psychotherapy interventions provided by a trained mental health professional (in our service, usually a psychologist) and oriented toward communication improvement and conflict solution in familiar contexts. This data is relevant since scientific literature demonstrated how the pandemic impacted the whole familiar system. Indeed, previous reports underlined that quarantine measures might influence depressive symptom severity among students and their family members (88). Studies focusing on the mental health of children, young adults and their parents showed considerable stressors that these populations perceived during the pandemic period. Children and adolescents were mainly stressed by the disruption of social life and important activities/events, whereas their parents were stressed by the uncertainty of the pandemic and the disease itself (89). These changes in habits suggest that specific risk factors for the development of psychological distress should be identified for both youths and their families in order to act on potentially modifiable stressors. Despite this, the readaptation of family therapy models to digital mental health settings, which was needed due to physical distancing protocols, required a huge effort, and several challenges were faced by both professionals and users (90). Indeed, the lack of adequate technology could represent a concern for families already coping with socio-economic problems before the pandemic due to the worsening of such problems in most cases (91).

Furthermore, the engagement with the therapist could become a concern for families that were not already in contact with the service in the “pre-COVID-19” period. This issue could explain the decrease in such interventions, possibly due to one or more members’ difficulties trusting the therapist and establishing a therapeutic alliance (92). A decreasing trend was not evidenced for social and educational interventions, usually requiring one-to-one relationships between social workers/professional educators and the user. This relationship does not happen in the context of a therapeutic process and does not undergo the rules of a psychotherapeutic setting, making it easier to adapt the intervention for a digital setting.

These considerations reinforce the need for integrated interventions in adolescents showing the onset of psychiatric symptoms during the pandemic (93). Integrated psychosocial interventions could avoid the detriments of more extended home-schooling periods, the loss of opportunities to meet peers, and the disruption of familiar daily routines (89). Accordingly, the finding concerning the reduction in access to residential facilities should be considered, even though the significance was low. This could be interpreted in consideration of significant challenges faced in youth residential care, where social distancing measures and the interruption of contacts with families of origin critically affected the possibility of providing integrative care (94).

Regarding substance use, in our study, we observed a reduction in opioid use in the “during-COVID-19” period of medium significance and, consequently, a reduction in the use of substitution therapy for drug addiction. This evidence could be related to the limitations produced by the lockdown measures during the pandemic period and confirms data from previous studies (95, 96).

However, our sample’s decrease in opioid consumption should be considered a part of a more complex, multi-facet situation. Indeed, due to reduced access to treatment and replacement pharmacology therapies and the lack of continuity in the intake of opioids, emergencies occurred more frequently in the pandemic period, as demonstrated by the increase in cases of opioid overdose (97).

Our study has limitations: first, the relatively small sample size may limit the generalizability of the findings. The issue is also due to the choice of a real-world setting relying on data from one service, since outpatient facilities dedicated to adolescents and young adults suffering from mental health problems are limited in our region. The sample size also hindered the possibility to perform further sub-analyses, e.g., stratifying subjects based on psychiatric diagnoses. Furthermore, it should be considered that data concerning the “during-COVID-19” period were collected during different pandemic phases, without, e.g., specifying whether new accesses happened during lockdown periods or not. To note, we could not analyze any increasing trends in the considered variables during the years preceding the COVID-19 outbreak, and the comparison between the pre-COVID-19 and the during-COVID-19 period assumed no increasing trends in the variables of interest.

Moreover, a specific psychopathological assessment was not systematically administered, and data collected in the usual clinical practice were instead used. This issue may limit the possibility to evaluate treatment response in the considered population. To this end, further studies should evaluate the outcomes of the administered interventions in youths suffering from psychological distress, particularly focusing on telepsychiatry and psychosocial treatments.

## Conclusion

Data from the present study suggest that health professionals should accurately screen youths for the presence of psychological distress, both those that already suffered from a psychiatric disorder and those manifesting such distress for the first time. Youths represent a high-risk population for the development of mental disorders, and these were demonstrated to increase during the COVID-19 pandemic and could be expected to rise in the post-pandemic era. The increase of specific psychopathological features in this vulnerable group after the COVID-19 outbreak suggests that pathways to care should be reinforced, and targeted interventions should be proposed to improve the mental health of adolescents and young adults. Particularly, clinicians should further promote the adaptation of mental health services to the emerging historical and social context, e.g., extensively rethinking services under a digital mental health perspective. Furthermore, the proposed interventions should include tailored pharmacological treatments that could help achieve symptomatologic remission and psychosocial interventions that would progressively lead youths toward a full-functional recovery.

## Data Availability Statement

The raw data supporting the conclusions of this article will be made available by the authors, without undue reservation.

## Ethics Statement

The studies involving human participants were reviewed and approved by the Ethics Committee of Umbria Region (protocol N° 23369/21/ON). Written informed consent to participate in this study was provided by the participants’ legal guardian/next of kin.

## Author Contributions

GM and GP conceived the idea and designed the study. GP and AS collected the data. GM performed the statistical analysis. GM, GP, FB, AM, NM, and MD’A wrote the original draft. SB, EM, LO, UV, and AT revised the whole manuscript. LS supervised the study during all its phases. All authors contributed to the article and approved the submitted version.

## Conflict of Interest

The authors declare that the research was conducted in the absence of any commercial or financial relationships that could be construed as a potential conflict of interest.

## Publisher’s Note

All claims expressed in this article are solely those of the authors and do not necessarily represent those of their affiliated organizations, or those of the publisher, the editors and the reviewers. Any product that may be evaluated in this article, or claim that may be made by its manufacturer, is not guaranteed or endorsed by the publisher.
